# Association Between Time to PSA Nadir, Radiologic Progression, and PSA Progression in mHSPC Patients Treated with Abiraterone or Enzalutamide

**DOI:** 10.3390/jcm15010386

**Published:** 2026-01-05

**Authors:** Ugur Ozkerim, Oguzcan Kinikoglu, Deniz Isik, Yunus Emre Altintas, Seval Ay Ersoy, Heves Surmeli, Hatice Odabas, Tugba Basoglu, Nedim Turan

**Affiliations:** Department of Medical Oncology, Health Science University, Kartal Dr. Lütfi Kirdar City Hospital, Istanbul 34865, Turkey; ogokinikoglu@yahoo.com (O.K.); dnz.1984@yahoo.com (D.I.); yunusaltintas1688@gmail.com (Y.E.A.); drsevalay@gmail.com (S.A.E.); hevessurmeli@hotmail.com (H.S.); odabashatice@yahoo.com (H.O.); basoglutugba@gmail.com (T.B.); turan.nedim@hotmail.com (N.T.)

**Keywords:** PSA kinetics, time to PSA nadir, metastatic hormone-sensitive prostate cancer, abiraterone, enzalutamide

## Abstract

**Background**: Time to prostate-specific antigen (PSA) nadir (TTN) has been proposed as an early indicator of treatment responsiveness in metastatic hormone-sensitive prostate cancer (mHSPC). However, its prognostic relevance in patients treated with next-generation androgen receptor pathway inhibitors (ARPIs), such as abiraterone or enzalutamide, remains incompletely defined. **Methods**: This retrospective cohort study included 147 patients with mHSPC treated with abiraterone or enzalutamide between 2019 and 2024. TTN, PSA kinetics, radiologic progression-free survival (rPFS), and PSA progression-free survival (PSA-PFS) were analyzed using Kaplan–Meier methods and multivariable Cox regression. TTN was evaluated both as a continuous variable and dichotomized at the cohort median (≤9 vs. >9 months). **Results**: TTN distributions were comparable between treatment groups (median 9.0 vs. 6.0 months, *p* = 0.197). Patients with a shorter TTN (≤9 months) experienced significantly longer median rPFS compared with those with longer TTN (>9 months) (10.7 vs. 7.95 months; *p* = 0.036). No significant association was observed between TTN and PSA-PFS (9.3 vs. 10.75 months; *p* = 0.34). In multivariable analysis, enzalutamide was independently associated with a reduced risk of radiologic progression compared with abiraterone (HR 0.622; 95% CI 0.441–0.877), whereas TTN was not an independent predictor. **Conclusions**: A shorter TTN was associated with improved radiologic outcomes, suggesting that rapid PSA suppression may reflect more favorable disease biology in patients receiving ARPI therapy. Although TTN showed limited value in predicting biochemical progression, it may serve as a simple and accessible biomarker for early risk stratification and tailoring follow-up intensity in mHSPC. Validation in larger, multicenter cohorts is warranted.

## 1. Introduction

Prostate cancer is among the most common male malignancies, and a considerable proportion of cases present with or evolve into metastatic hormone-sensitive prostate cancer (mHSPC). Treatment in this disease condition involves androgen deprivation therapy (ADT) together with next-generation androgen receptor pathway inhibitors (ARPIs) like abiraterone acetate and enzalutamide which have effectively increased patient survival and disease control although treatment responses are not uniform across the patient populations [1,2,3]. The knowledge of biomarkers which indicate the outcome of the treatment is thus critical in streamlining the therapeutic approaches of different clinical environments.

The kinetics of prostate-specific antigen (PSA), specifically, the values of PSA nadirs and time to PSA nadirs (TTN) are becoming clinically significant parameters to assess tumor response in hormonal suppression. Previous studies have demonstrated that TTN is an independent predictor of overall survival in patients undergoing ADT therapy of metastatic hormone-sensitive disease, indicating that it may be used as a surrogate biomarker [4,5]. On the same note, both TTN and nadir PSA have been implicated in patterns of progression and resistance to treatments across multiple treatment settings, including ARPI therapy, with rapid biochemical progression possibly indicating unique tumor biology in comparison to slow suppression of PSA [6,7,8].

Recent real-world studies in the era of treatment intensification have continued to evaluate TTN (time to PSA nadir) and related PSA-kinetic endpoints as prognostic markers in mHSPC, including cohorts treated with ARPIs. However, the reported associations between TTN and clinical outcomes appear heterogeneous across datasets, likely reflecting differences in patient risk profiles, treatment strategies, and follow-up intensity. These findings highlight the need for population-specific validation of TTN as a practical biomarker in routine ARPI-treated mHSPC care [9].

The TTN prognostic value of patients receiving either abiraterone or enzalutamide treatment in particular is not fully understood despite these findings. Some of the studies have shown that PSA trajectory in the ADT or ARPI therapy can predict the degree of treatment response, time to progression, or progression to castration-resistant disease, although the findings have been inconsistent among cohorts, especially when stratified by metastatic volume, disease biology, or modalities of treatment [6,7,8]. Moreover, it seems that the PSA suppression dynamics can vary based on the metastasis load, initial tumor features and treatment regimens, which complicates the interpretation of PSA dynamics in clinical practice [3,10].

The heterogeneity in PSA kinetics among mHSPC patients receiving ARPIs has been demonstrated in Asian and European populations. In Turkey, real-world mHSPC management may be influenced by differences in access to imaging follow-up, variability in referral pathways to tertiary centers, and heterogeneous baseline disease burden at presentation. These factors may contribute to population-specific PSA monitoring patterns and timing of TTN estimation in routine practice [8].

**Figure 1:**
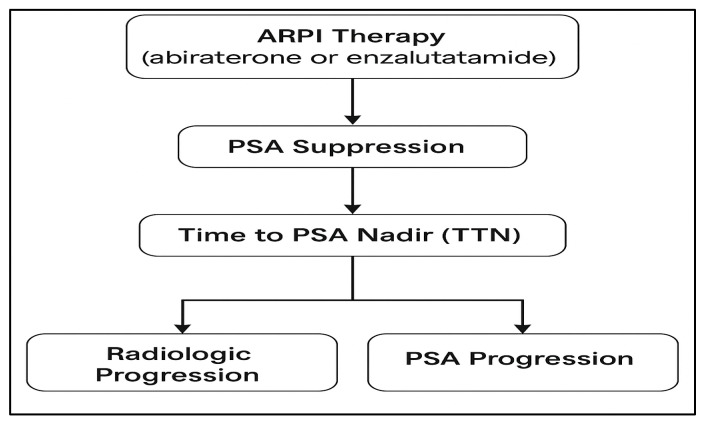
Theoretical model that demonstrates the interconnection between ARPI therapy (abiraterone or enzalutamide), PSA suppression, time to PSA nadir (TTN), and clinical outcomes of radiologic and PSA-based progression. Arrows indicate the sequential relationship between ARPI therapy, PSA suppression dynamics, TTN, and subsequent clinical outcomes.

Figure 1 gives a conceptual overview of how ARPI therapy, PSA suppression dynamics, TTN, and downstream clinical endpoints (radiologic and PSA-based progression) interact and is a mechanistic and clinical rationale as to why TTN needs to be evaluated as a prognostic biomarker in such a population.

The current research intends to examine:

(1) TTN variation in patients receiving abiraterone and enzalutamide in Turkey;
(2) the correlation between TTN and radiologic progression;
(3) the correlation between TTN and the development of PSA;
(4) the independent predictive TTN with clinical covariates using multivariate models.

This study, combining the dynamics of PSA with radiologic and biochemical outcomes, bridges a gap in the important evidence base and forms part of the emerging literature on the biomarker-based treatment of mHSPC.

## 2. Methodology

### 2.1. Study Design

This study employed a retrospective cohort design, using real-world clinical data from patients with metastatic hormone-sensitive prostate cancer (mHSPC) treated with abiraterone acetate or enzalutamide between 2019 and 2024. Retrospective cohort designs are widely used in prostate cancer research to evaluate biomarker behavior, PSA kinetics, treatment responses, and survival outcomes in routine clinical settings [8,9]. The study methodology adhered to the STROBE guidelines to ensure transparent and high-quality reporting.

**Figure 2:**
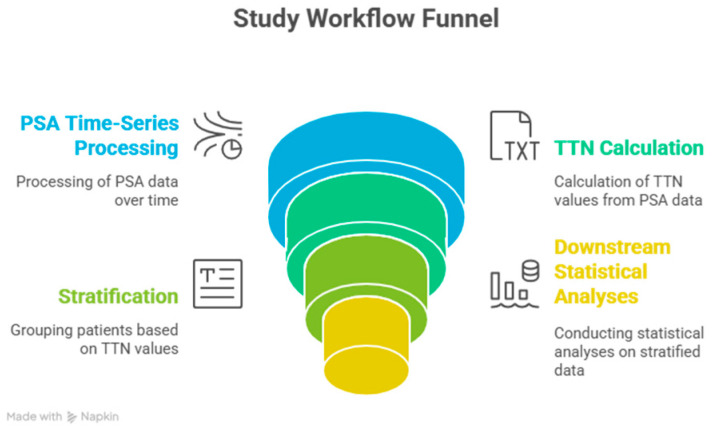
Patient selection, PSA time-series processing, TTN computation, stratification and downstream statistical analysis Study workflow Reviewed.

Figure 2 shows a schematic representation of the study workflow, which includes the processes of patient selection, data extraction, processing of PSA series, TTN, and survival modelling.

### 2.2. Study Population

The data to be analyzed consisted of 147 patients who were diagnosed with mHSPC and were treated during 2019 and 2024. The inclusion criteria were as follows: (i) patients had to have histologic-proven prostate adenocarcinoma; (ii) patients had to have metastatic disease at the time of initial diagnosis or systemic progression after local therapy; (iii) patients needed to receive abiraterone acetate or enzalutamide with ADT as the initial treatment; and (iv) patients had to have adequate PSA and imaging follow-up to calculate TTN, radiologic progression-free survival (rPFS), and PSA progression. Other real-life and prospective prostate cancer studies that have used similar inclusion frameworks have been conducted to test the PSA-based biomarkers and treatment response [3,8].

**Figure 3:**
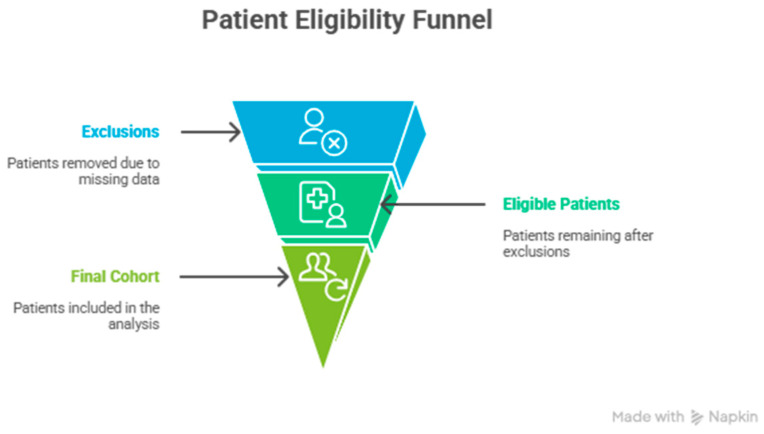
Flowchart of patient eligibility including, excluding, and forming of final analytic cohort (N = 147).

The exclusion criteria were the following: missing radiologic assessment; incomplete PSA time-series; prior systemic treatment of metastatic disease (except ADT); or lack of survival outcomes. Finally, out of the total number of patients (147), all of them met the requirements and were included in the analysis. Figure 3 is a summary of cohort derivation.

### 2.3. Ethics Approval

This study was conducted in accordance with the Declaration of Helsinki and was approved by the Kartal Dr. Lütfi Kırdar City Hospital Scientific Research Ethics Committee (Approval No: 2025/010.99/12/11, dated 24 January 2025). Since the study involved fully de-identified retrospective clinical data obtained from routine practice, the requirement for informed consent was waived by the ethics committee in accordance with national regulations, including the Regulation on Clinical Research (Official Gazette No. 28617, 13 April 2013, Article 2.2). All patient information accessed, stored, and analyzed remained within the scope of the approved protocol.

### 2.4. Variables and Definitions

#### 2.4.1. Time to PSA Nadir (TTN)

TTN was defined as the time interval (in months) between treatment initiation and the lowest recorded absolute PSA value during follow-up. PSA nadir was initially identified algorithmically as the minimum PSA value within each patient’s longitudinal PSA measurements and was subsequently verified by clinical review to ensure consistency with routine oncologic assessment. TTN is a well-established dynamic biomarker in prostate cancer, with prognostic associations reported for overall survival and radiologic outcomes across studies evaluating both androgen deprivation therapy and ARPI-based treatment strategies [4,11]. TTN was evaluated both as a continuous variable and as a categorical variable using the cohort median (9 months) to define short (≤9 months) and long (>9 months) TTN.

The optimal TTN cut-off value was determined using receiver operating characteristic (ROC) curve analysis, with radiologic progression as the outcome of interest. The ROC-derived threshold corresponded to 9 months, which also approximated the cohort median and allowed balanced group sizes for comparative analyses.

PSA measurements were obtained at variable intervals in accordance with real-world clinical practice, typically ranging from monthly to every three months. This variability reflects routine follow-up patterns in daily oncology care and did not preclude reliable determination of TTN.

To address potential bias related to PSA sampling frequency and the time-dependent nature of TTN, additional sensitivity analyses using a landmark approach were performed, as described in Section 2.

#### 2.4.2. PSA Nadir

The lowest level of absolute PSA achieved during ARPI-based therapy was considered as PSA nadir. PSA nadir has demonstrated prognostic importance in both hormone-sensitive and castration-resistant prostate cancer in terms of absolute nadir levels and the time required to reach these levels [11,12].

Radiologic progression-free survival (rPFS) was defined as the time from initiation of ARPI therapy to radiologic disease progression, assessed using CT, MRI, or bone scintigraphy according to standard clinical practice and internationally recognized imaging principles. Radiologic progression was determined based on routine radiology and nuclear medicine reports, considering the emergence of new metastatic lesions or unequivocal progression of existing disease, consistent with RECIST-based oncologic decision-making, without formal protocol-driven measurements. Patients without documented progression at last follow-up were censored at their most recent imaging date [5,13].

Missing data were limited to a small number of patients and primarily involved baseline clinical and laboratory variables. All included patients had regular and sufficiently frequent PSA follow-up, allowing reliable determination of time to PSA nadir (TTN). Radiologic progression-free survival (rPFS) could be assessed in all patients included in survival analyses. Given the low proportion and non-systematic nature of missing baseline data, no imputation methods were applied, and analyses were conducted using a complete-case approach.

#### 2.4.3. PSA Progression (PSA-PFS)

PSA progression-free survival (PSA-PFS) was defined as the time from initiation of ARPI therapy to biochemical progression. PSA progression was defined as an increase of ≥25% and ≥2.0 ng/mL above the PSA nadir, confirmed by a second measurement at least 3 weeks later, or a ≥25% increase from baseline if no decline from baseline was observed. Patients without PSA progression at last follow-up were censored at the date of their most recent PSA measurement [14].

#### 2.4.4. Clinical Covariates

The following baseline variables were incorporated because they were known to be prognostic variables in metastatic prostate cancer:

- Age;
- Baseline PSA;
- Gleason score/Grade Group;
- Metastatic load (high-volume vs. low-volume);
- PNI category (low vs. high), which is congruent with previous biomarker-based studies [15].

### 2.5. Data Extraction and Preprocessing

Institutional electronic medical records were used to extract PSA measurements, imaging schedules, biochemical parameters, and demographic data. Data preprocessing included standardization of PSA time series into time-aligned sequences, calculation of TTN and PSA nadir values, and derivation of survival endpoints. Missing data were handled according to best practices commonly applied in real-world oncology studies, ensuring that no imputation procedures distorted survival event definitions or the time-to-event structure [16].

### 2.6. Statistical Analysis

#### 2.6.1. Descriptive Statistics

Continuous variables: Means, median, standard deviations and interquartile ranges were used to present the summary of the continuous variables; Categorical variables: frequencies and percentages were used to present the summary of the categorical variables. The independent-samples *t*-tests or Mann–Whitney U tests were used to compare the groups of abiraterone- and enzalutamide-treated patient with the continuous variables and chi-square tests with categorical variables, respectively. Baseline demographic and clinical characteristics used for group comparisons and multivariable modeling are summarized in Table 1.

#### 2.6.2. TTN Analyses

TTN was compared between treatment groups and across PNI categories. TTN was also stratified using the cohort median (≤9 vs. >9 months) to evaluate differences in radiologic progression and PSA progression, consistent with methodologies used in prior TTN biomarker studies [4,11].

#### 2.6.3. Time-to-Event Analyses

Kaplan–Meier survival curves were generated for rPFS and PSA-PFS, and differences between TTN groups were assessed using the log-rank test. Kaplan–Meier analysis is a standard approach for evaluating survival outcomes in prostate cancer research [12,13]. Multivariable Cox proportional hazards regression was then performed to identify independent predictors of rPFS, assuming proportional hazards between covariates and the outcome.

Multivariable Cox models were used to evaluate independent predictors of rPFS. Covariates included:

- TTN (continuous or categorical);
- Type of treatment (abiraterone or enzalutamide);
- Age;
- Baseline PSA;
- PNI category.

Hazard ratios (HRs) with 95% confidence intervals were reported. The evaluation of model assumptions was based on Schoenfeld residuals and proportionality diagnostics [15].

Given the non-randomized nature of treatment allocation, multivariable Cox regression was used to adjust for key baseline clinical and disease-related covariates known to influence radiologic outcomes in mHSPC. Baseline PSA values demonstrated a right-skewed distribution and were therefore summarized using medians and interquartile ranges, consistent with standard practice in prostate cancer studies.

#### 2.6.4. Significance Threshold

All statistical tests were two-sided, with a significance level set at α = 0.05. Statistical analyses were performed using IBM SPSS Statistics version 27 and were verified by recalculation.

## 3. Results

### 3.1. Baseline Characteristics of the Study Population

The study cohort consisted of 147 Turkish patients with metastatic hormone-sensitive prostate cancer, including 74 patients treated with abiraterone and 73 treated with enzalutamide. Baseline demographic and clinical characteristics were generally well balanced between treatment groups, with no statistically significant differences observed in age, baseline PSA levels, Gleason/Grade Group distribution, metastatic burden, or PNI category (Table 1). These baseline distributions were consistent with those reported in prior real-world cohorts evaluating biomarkers and therapeutic outcomes in hormone-sensitive metastatic prostate cancer [3,8]. Although minor numerical differences in ECOG performance status distribution were observed, these differences did not reach statistical significance.

Median baseline PSA levels were relatively high and demonstrated substantial heterogeneity, reflecting variability in disease burden at diagnosis. Approximately one-third of patients presented with high-volume metastatic disease, consistent with epidemiologic patterns reported in recent mHSPC cohorts [12]. PNI category (high vs. low) was similarly distributed across treatment groups, minimizing potential confounding in analyses evaluating interactions between TTN and clinical covariates.

### 3.2. Kinetics of PSA and Time to PSA Nadir (TTN)

Clustering of PSA kinetics was observed to be specific at nadir intervals of 6, 9 and 12 months indicating the presence of structured practices in routine clinical settings regarding follow-up. The average TTN across the whole group of the cohort was equal to 8.67 months and was 9 months in the middle, which is the natural dichotomization indicator to use thereafter.

In comparison of treatment groups:

- Abiraterone: TTN = 8.96; median = 9.0.
- Enzalutamide: median TTN = 6.0; mean = 8.38 months.

The group difference was not statistically significant (*p* = 0.197), which reflected patterns reported in previous analyses indicating broadly similar early PSA suppression between ARPIs in hormone-sensitive disease [17,18]. However, despite the lack of statistical significance, the observed numerical separation in median TTN between treatment groups (6 months for enzalutamide vs. 9 months for abiraterone) may still be clinically relevant, potentially reflecting subtle differences in early PSA suppression dynamics. Given the real-world design and limited sample size, this observation should be considered hypothesis-generating and warrants confirmation in larger cohorts. With the median cutoff of 9 months:

- Short TTN (≤9 months): 95 patients;
- Long TTN (>9 months): 52 patients.

Homogeneity was exhibited in the follow-up timing as patients with long TTN reached nadir uniformly at 12 months. These group comparisons and distributions are summarized in Table 2.

### 3.3. Association Between TTN and Radiologic Progression-Free Survival (rPFS)

The relationship between TTN and radiologic progression-free survival (rPFS) was evaluated using correlation analysis. Radiologic progression occurred in 146 of 147 patients during follow-up. The mean rPFS was longer in patients with short TTN (≤9 months) compared with those with long TTN (>9 months) (10.39 vs. 8.97 months). Similarly, median rPFS was higher in the short TTN group than in the long TTN group (10.7 vs. 7.95 months). This difference was statistically significant (*p* = 0.036), indicating an unadjusted association between shorter TTN and improved radiologic outcomes in this cohort.

This pattern may reflect population-specific disease and treatment characteristics in this real-world Turkish cohort. While some Western trial-based cohorts have reported favorable outcomes with longer TTN, the observed association in our study aligns with findings from several Asian real-world datasets, suggesting biological and clinical heterogeneity in ARPI responsiveness and PSA suppression dynamics across populations [3,4,17]. These differences may be influenced by baseline disease burden, treatment intensity, and follow-up practices in routine clinical care.

**Figure 4:**
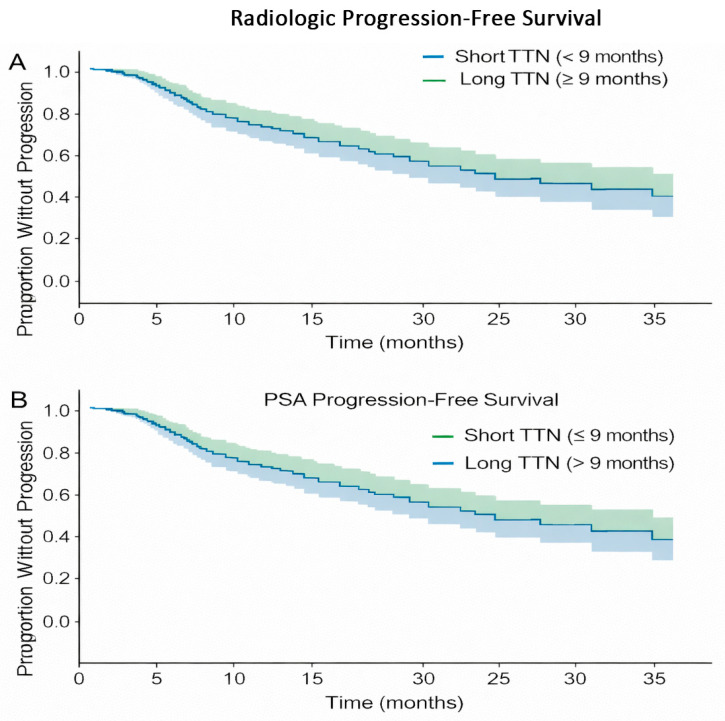
Kaplan–Meier survival curves according to time to PSA nadir (TTN ≤ 9 vs. > 9 months) in patients with metastatic hormone-sensitive prostate cancer treated with androgen receptor pathway inhibitors (ARPIs), showing (**A**) radiologic progression-free survival (rPFS) and (**B**) PSA progression-free survival (PSA-PFS). The solid line represents the estimated Kaplan–Meier survival curve, while the shaded areas represent the 95% confidence intervals. The blue and green shaded areas indicate the lower and upper bounds of the confidence interval, respectively.

A weak negative correlation between TTN and rPFS (r = −0.17) further suggests that prolonged time to biochemical suppression does not necessarily translate into radiologic benefit in this population. Kaplan–Meier curves comparing rPFS across TTN groups are presented in Figure 4.

### 3.4. Relationship Between TTN and PSA Progression-Free Survival (PSA-PFS)

All patients experienced PSA-based progression (event rate = 100%), enabling time-to-event analyses without censoring. TTN demonstrated a very weak correlation with PSA-PFS (r = 0.05), indicating minimal association between time to PSA nadir and biochemical progression.

**Figure 5:**
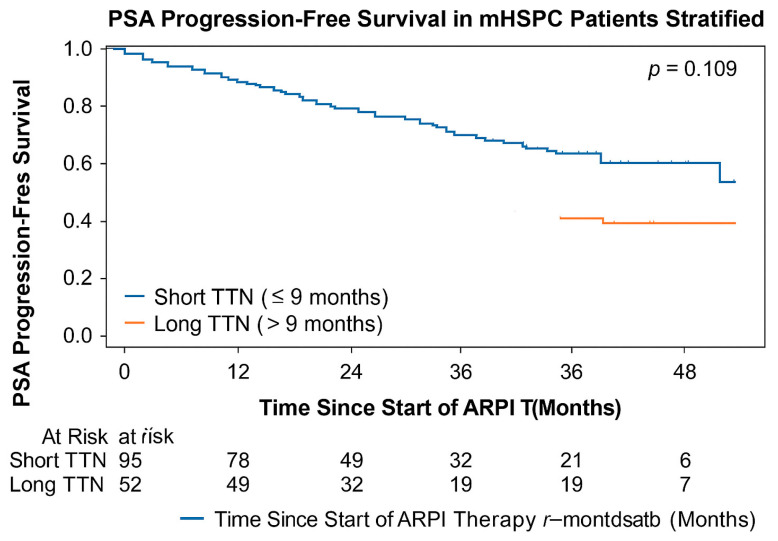
Kaplan–Meier curves of progression-free survival of PSA depending on the TTN category.

Median PSA-PFS was 9.3 months in patients with short TTN (≤9 months) and 10.75 months in those with long TTN (>9 months), with no statistically significant difference between groups (log-rank *p* = 0.109). As illustrated in Figure 5, Kaplan–Meier curves for PSA-PFS largely overlapped across TTN categories, supporting the absence of a meaningful association between TTN and biochemical progression. This finding is consistent with prior evidence suggesting that PSA kinetics and biochemical relapse may diverge during the hormone-sensitive phase due to differences in disease burden and intratumoral heterogeneity [19,20].

In order to support these results, the summary statistics of PSA-PFS between groups are given in Table 3.

### 3.5. Effects of the Treatments and Multivariable Cox Regression

The type of treatment was shown to be an important independent predictor in the multivariate Cox proportional hazards model of rPFS:

Enzalutamide compared to Abiraterone:

- HR = 0.622;
- 95% CI: 0.441–0.877;
- *p* = 0.007.

This corresponds to an approximate 38% relative reduction in the risk of radiologic progression, independent of TTN and other covariates.

**Figure 6:**
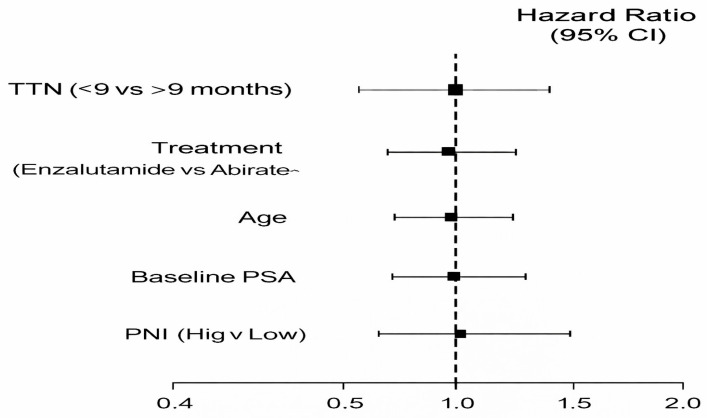
Summary of Forest plot of Cox regression hazard ratios of predictors of radiologic progression.

The other covariates considered (age, baseline PSA, PNI category) failed to achieve statistical significance-which is also in line with previous models which focused more on disease biology and ARPI sensitivity as the key determinants of early radiologic results [21,22]. All model coefficients are summarized in Table 4 and the forest plot is shown in Figure 6. In a pre-specified landmark analysis at 9 months, TTN was not retained as an independent predictor of radiologic progression-free survival, whereas the treatment effect of enzalutamide remained consistent.

## 4. Discussion

In this real-world cohort of patients with metastatic hormone-sensitive prostate cancer treated with ARPIs, TTN was not identified as an independent predictor of radiologic progression-free survival in multivariable analysis. However, a shorter TTN was consistently associated with improved rPFS, suggesting that early PSA suppression dynamics may still carry clinically relevant prognostic information.

Using real-world data from a Turkish mHSPC population treated with abiraterone or enzalutamide, the present study provides population-specific insights into the prognostic role of PSA kinetics, particularly TTN, a biomarker with heterogeneous implications across global cohorts. Our findings can be summarized as follows: (i) TTN did not differ significantly between treatment groups, (ii) shorter TTN was associated with improved radiologic progression-free survival, and (iii) TTN was not predictive of PSA-based progression. Collectively, these results highlight the complex interplay between PSA suppression dynamics, treatment biology, and clinical outcomes in mHSPC.

The fact that TTN did not differ significantly between the group of patients treated with abiraterone versus those with enzalutamide agrees with the previous data that both ARPIs cause strong androgen receptor pathway inhibition but possibly with a different mechanism of downstream PSA decline [23]. Though enzalutamide, by invoking direct androgen receptor inhibition, and abiraterone, by blocking androgen biosynthesis, have early biochemical patterns which converge, especially during the hormone sensitive phase, when tumor androgen dependence prevails.

Interestingly, the data set proved that shorter TTN (≤9 months) was strongly linked with longer rPFS, which should be interpreted in detail. Certain previous experiments with mixed mHSPC and mCRPC populations indicated that long-term TTN could be due to regulated tumor dynamics, or more profound inhibition of androgen-stimulated signaling [24,25]. But other studies- especially in Asian and Eastern European studies- have shown the opposite, i.e., brisk bio-chemical response to be associated with improved radiologic and survival results, possibly because of biological disparities in tumor androgen receptor modification and pharmacodynamic effects of ARPIs [17,26]. Our findings are more consistent with the latter view, suggesting that rapid PSA decline in this Turkish cohort may reflect an inherent sensitivity to ARPI therapy.

A possible reason that can be attributed to positive results of shorter TTN is connected to the tumor burden and disease phenotype. In practice, it has been demonstrated that patients with high-volume disease, visceral metastases, or aggressive biological behavior tend to have a delayed or a slowed down PSA decline despite ARPI therapy [22]. Conversely, those patients whose tumors are more androgen-dependent or of less genomically complex tumors report quick suppression of PSA, which translates to a better radiologic disease control. Even though the metastatic volume was equal between the arms of the treatment groups in our cohort, the interpatient variation in the expression of the androgen receptor, lineage plasticity, or clonal heterogeneity-measures that cannot be identified in standard clinical data sets-could be a partial cause of the existing TTN-rPFS relationship.

This observation that TTN was not a predictor of PSA progression (PSA-PFS) only tells of the subtle significance of PSA kinetics. Subtle intratumoral changes, secondary resistance mechanisms, or delayed biochemical escape is also common in biochemical progression, which is not necessarily associated with radiologic changes [19,27]. This lack of association between PSA kinetics and biochemical relapse has been reported in numerous studies that have investigated ARPI-treated populations, and the insufficient sensitivity of PSA alone to detect early resistance in hormone-sensitive disease [28]. In this way, even though TTN might be relevant to early disease biology, as is pertinent in relation to radiologic response, TTN might not sufficiently represent pathways controlling PSA relapse, especially when discordant biochemical and radiologic patterns are caused by ARPI-induced oncogenic reprogramming.

Multivariate Cox model revealed that enzalutamide had a 38 percent decrease in radiologic progression risk than abiraterone with no impact of TTN and other clinical covariates. These results are consistent with various clinical observations and meta-analytic data that enzalutamide can provide greater disease control in certain subsets of mHSPC which could be because it directly interferes with androgen receptor signaling [11,29]. The fact that PNI, age, and baseline PSA had no significant effects is consistent with the trends in the larger literature, in which biochemical and static clinical variables tend to have weaker prognostic impact in ARPI-augmented therapy, particularly in the early stages of the disease course [18,21].

Collectively, these results highlight the fact that TTN cannot be viewed as a universal prognostic variable, but, instead, as a situational factor the meaning of which is limited to ethnic, biological, and treatment-based subgroups. In the Turkish population under investigation in this paper, the rapid achievement of PSA nadir seems to be associated with better radiologic results which is in line with models where early response of ARPI indicates less tumor adaptability and greater dependence on androgens. Further characterization of these patterns through future integration of genomic profiling including repair alterations of DNA, AR-V7 status and clonal phylogeny could allow more personalized interpretation of PSA kinetics in mHSPC.

From a clinical perspective, TTN represents a simple, inexpensive, and widely accessible biomarker that may help inform follow-up strategies in routine practice. In this Turkish real-world cohort, rapid achievement of PSA nadir appears to reflect greater androgen dependence and more favorable radiologic outcomes, supporting the potential role of TTN as an early risk stratification tool. Patients achieving a short TTN could potentially be reassured and managed with standard or less intensive imaging schedules, whereas those with prolonged TTN may warrant closer radiologic surveillance or earlier consideration of treatment modification. These observations align with emerging biomarker-driven approaches to treatment personalization in prostate cancer and should be validated in prospective studies [19,30].

## 5. Conclusions

This study demonstrates that time to PSA nadir (TTN) is a clinically accessible and early prognostic marker in patients with metastatic hormone-sensitive prostate cancer treated with abiraterone or enzalutamide. Although TTN distributions were similar between treatment groups, a shorter TTN was associated with improved radiologic progression-free survival, suggesting that rapid biochemical response may reflect more favorable disease biology. In contrast, TTN showed no meaningful association with PSA-based progression, underscoring the complexity of PSA kinetics in the hormone-sensitive setting.

Enzalutamide remained an independent predictor of improved radiologic outcomes irrespective of baseline clinical characteristics or PSA kinetics, supporting its effectiveness in routine clinical practice.

Overall, TTN represents a low-cost and readily obtainable biomarker that may assist in early risk stratification and treatment monitoring in real-world mHSPC care. Further validation in multicenter prospective cohorts, together with integration of molecular and genomic profiling, is warranted to better define the role of TTN in personalized risk stratification.

## 6. Limitations

This study has several limitations. First, its retrospective design introduces potential selection bias, incomplete documentation, and non-standardized PSA and imaging intervals common to real-world datasets [13]. Second, the cohort originates from a single national setting, which may limit generalizability, as biological, ethnic, and access-related differences have been shown to influence ARPI responsiveness and PSA suppression patterns across populations [8]. Third, radiologic progression assessments were based on routine clinical practice rather than protocol-driven schedules, which may affect the timing of progression detection, consistent with prior real-world observations [12]. Fourth, TTN was dichotomized using the cohort median (9 months), a data-driven cut-point that may not represent a biologically validated threshold, and TTN distributions may differ across populations due to variations in disease burden, treatment sequencing, and tumor biology [15]. Finally, genomic, molecular, and advanced imaging biomarkers were not available; factors such as AR-V7 expression, DNA damage repair alterations, and molecular signatures are known to influence ARPI sensitivity and may partly explain discordance between PSA kinetics and radiologic progression [31,32]. Prospective multicenter studies with standardized follow-up and integrated biomarker profiling are needed to validate these findings.

In addition, PSA kinetics in real-world practice may be influenced by variability in testing intervals and follow-up schedules, which should be considered when interpreting TTN and other PSA-based parameters. As treatment allocation was not randomized, some degree of residual confounding cannot be fully excluded despite multivariable adjustment.

## Figures and Tables

**Table 1:** Baseline characteristics of patients with metastatic hormone-sensitive prostate cancer treated with abiraterone or enzalutamide (N = 147).

Characteristic	Abiraterone (*n* = 74)	Enzalutamide (*n* = 73)	Total (N = 147)
Demographics			
Age, years	68.9 ± 7.4 (48–86)	67.8 ± 8.3 (50–85)	68.4 ± 7.9 (48–86)
Follow-up duration, months	25.1 (16.4–39.8) (2.1–82.0)	28.9 (18.2–41.7) (3.0–85.4)	27.0 (17.1–40.6) (2.1–85.4)
Disease burden/biology			
Metastatic volume (CHAARTED)			
- Low volume	27 (36.5%)	30 (41.1%)	57 (38.8%)
- High volume	47 (63.5%)	43 (58.9%)	90 (61.2%)
Visceral metastasis	6 (8.1%)	3 (4.1%)	9 (6.1%)
Bone metastasis present	51 (68.9%)	47 (64.4%)	98 (66.7%)
ISUP/Gleason Grade Group			
- Grade Group 1	1 (1.4%)	2 (2.7%)	3 (2.0%)
- Grade Group 2	2 (2.7%)	1 (1.4%)	3 (2.0%)
- Grade Group 3	5 (6.8%)	7 (9.6%)	12 (8.2%)
- Grade Group 4	8 (10.8%)	13 (17.8%)	21 (14.3%)
- Grade Group 5	58 (78.4%)	50 (68.5%)	108 (73.5%)
Clinical status			
ECOG Performance Status			
- ECOG 0	14 (18.9%)	28 (38.4%)	42 (28.6%)
- ECOG 1	12 (16.2%)	16 (21.9%)	28 (19.0%)
- ECOG ≥ 2	48 (64.9%)	29 (39.7%)	77 (52.4%)
Prognostic Nutritional Index (PNI)			
- High PNI	51 (68.9%)	45 (61.6%)	96 (65.3%)
- Low PNI	23 (31.1%)	28 (38.4%)	51 (34.7%)
PSA-related variables			
Baseline PSA, ng/mL	82.0 (34–190) (0.5–1820)	75.0 (29–171) (0.4–1650)	78.0 (32–182) (0.4–1820)
PSA sampling intensity			
PSA measurements per patient	10 (8–13)	11 (8–14)	10 (8–13)

Values are presented as mean ± standard deviation (SD), median (interquartile range, IQR), or number (%), as appropriate. No statistically significant differences were observed between the abiraterone and enzalutamide groups across baseline variables.

**Table 2:** PSA kinetics and distribution of TTN among groups of treatment and TTN categories.

PSA Kinetics Variable	Abiraterone (*n* = 74)	Enzalutamide (*n* = 73)	Total (N = 147)	*p*-Value
Mean TTN (months)	8.96	8.38	8.67	0.197
Median TTN (months)	9.0	6.0	9.0	—
Short TTN (≤9 months)	46 (62.2%)	49 (67.1%)	95 (64.6%)	>0.05
Long TTN (>9 months)	28 (37.8%)	24 (32.9%)	52 (35.4%)	>0.05

**Table 3:** PSA progression-free survival (PSA-PFS) outcomes according to TTN categories.

PSA-PFS Variable	Short TTN (≤9 Months) (*n* = 95)	Long TTN (>9 Months) (*n* = 52)	*p*-Value
Median PSA-PFS (months)	9.30	10.75	0.109
PSA Progression Events (*n*)	95 (100%)	52 (100%)	—

**Table 4:** Multivariate Cox regression analysis of predictors of radiologic progression-free survival.

Variable	Hazard Ratio (HR)	95% Confidence Interval (CI)	*p*-Value
TTN (≤9 months vs. >9 months)	HR: 1.10	0.78–1.56	>0.05
Treatment Type (Enzalutamide vs. Abiraterone)	0.622	0.441–0.877	0.007
Age (years)	1.01	0.99–1.03	>0.05
Baseline PSA (ng/mL)	1.00	0.99–1.00	>0.05
PNI Category (High vs. Low)	1.15	0.82–1.60	>0.05

## Data Availability

The clinical data used in this study were obtained from institutional medical records in accordance with ethics approval. Because the dataset contains identifiable health information that cannot be shared publicly, it is not available for open distribution. However, de-identified versions of the relevant variables used for statistical analyses can be provided by the corresponding author upon reasonable request, in compliance with institutional and national data protection regulations.
